# From Passive to Active: Self‐Propelled Colloids in Coatings Formulation and Film Formation

**DOI:** 10.1002/smll.73903

**Published:** 2026-05-31

**Authors:** Karnika Singh, Jan Cammann, Luka Burduli, Edgar Espinosa Rodriguez, Franck D'Agosto, Muriel Lansalot, Ignacio Martin‐Fabiani

**Affiliations:** ^1^ Department of Materials Loughborough University Loughborough UK; ^2^ Interdisciplinary Centre for Mathematical Modelling and Department of Mathematical Sciences Loughborough University Loughborough UK; ^3^ School of Science Constructor University Bremen Germany; ^4^ Catalysis, Polymerization, Processes and Materials (CP2M) Universite Claude Bernard Lyon 1 Villeurbanne France

**Keywords:** active matter, assembly, coatings, colloids, formulation, interfaces

## Abstract

Coatings are widely used in protective and functional applications but are fundamentally limited by the passive nature of their formulation ingredients. This leads to a critical lack of control over the spatial distribution of ingredients and prevents the optimization of key functional properties. Here, we demonstrate the use of active, self‐propelled constituents in coatings formulations. By introducing active Janus colloids (JCs), we show how they overcome sedimentation and chemical gradients to accumulate at both top and bottom coating interfaces. We find that balancing the timescales of fuel depletion and evaporation‐induced assembly is key to control the JCs distribution within the dried coating. JCs at the top coating surface have an orientational bias, with the sub‐equatorial orientation being the most common. While our study is a proof‐of‐concept demonstration on a model system, it establishes a framework for harnessing active Janus colloids to influence coating microstructure. This work could lay the foundation for future studies developing functional coatings with tuned microstructure enabled by orientation‐biased active JCs.

## Graphical abstract

Self‐propelled Janus colloids are introduced into coating formulations to overcome passive transport limitations during drying. Careful balance of particle activity and evaporation timescales results in their accumulation at both coating interfaces as well as an orientational bias at the top surface. A new route to control coating microstructure harnessing active matter principles is demonstrated.

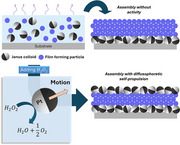

## 1 Introduction

Coatings are ubiquitous. They are found on almost every object and surface we interact with daily. They serve both decorative and functional purposes such as protection from wear, corrosion [1, 2, 3], and fouling [4, 5, 6], or providing properties like electrical conductivity [7] or photocatalytic activity [8]. Across most applications, a key limitation to coating performance is the lack of control over the final spatial distribution of functional ingredients within the film. Recent advances have shown how size segregation processes occurring during the drying of colloidal blends can be harnessed to drive smaller particles to the top of the coating [9, 10, 11, 12, 13]. When these surface‐enriched particles are functional, they provide enhanced performance, e.g., improved antibacterial activity [8] or greater abrasion resistance [14], by being concentrated precisely where their action is most effective. However, it remains unclear how this segregation process could be translated to the more complex formulations used in commercial coatings. Studies show how the addition of rheology modifiers give rise to complex behavior and a range of coating microstructure scenarios [15, 16] even before considering the wide range of other formulation ingredients typically present in coatings. Structures other than small‐on‐top have been reported, such as small‐large‐small [17], but these occur within a narrow range of systems and environmental conditions. The work we present here serves as a proof of concept on how to harness self‐propelled ingredients in coating design. For the first time, we introduce active ingredients into coatings formulations and demonstrate how they can be used to control the resulting microstructure.

Active colloids are synthetic microswimmers that consume energy from their surroundings to move forward. In systems where hydrogen peroxide serves as the fuel, platinum (Pt)‐coated JCs propel by self‐diffusiophoresis. The catalytic Pt hemisphere decomposes $H_{2}O_{2}$, producing spatially asymmetric chemical gradients that generate a phoretic slip velocity along the particle surface:

$$H_{2}O_{2}\overset{Pt}{⟶}H_{2}O+\frac{1}{2}O_{2}$$

The resulting solute gradients break symmetry and drive the propulsion of JCs. Unlike passive particles that remain at thermal equilibrium, active colloids operate far from equilibrium, giving rise to collective behaviors and self‐organization phenomena [18, 19, 20, 21]. The introduction of these self‐propelling particles into coating formulations has the potential to overcome limitations posed by passive ingredients. Their propulsion can compete with convective and diffusive transport, such as those generated by undesired coffee‐ring effects [22]. Importantly, active particles generate their own local gradients and forces, making their spatial distribution more reliable and less dependent on the formulation specifics [23]. Active‐passive colloidal mixtures have been extensively explored in liquid suspensions to study clustering, transport, and emergent collective dynamics [24, 25, 26, 27, 28, 29, 30, 31]. Even a small fraction of active particles can drive phase separation, dynamic clustering, and enhanced mobility of passive components through a combination of phoretic and hydrodynamic interactions  [24, 25, 26]. Theoretical and experimental studies have shown that such mixtures exhibit activity‐induced demixing  [24, 25], long‐range hydrodynamic and effective interactions between passive inclusions  [27, 28], and enhanced diffusion or directed transport of passive tracers in active baths  [26, 29]. Recent advances in active matter have further expanded the understanding of how self‐propelled particles behave in complex, time‐evolving, and structured environments. Studies have shown that structured energy landscapes can stabilise collective states of active particles [32], while environmental memory effects can significantly influence their collective dynamics and group formation [33]. More recent work has also explored how interactions, confinement, and dynamically evolving surroundings affect the organization and transport of active colloids [34, 35]. These developments highlight that active particles are highly sensitive to their local environment, including interfaces, confinement, and time‐dependent conditions. However, to our knowledge, active matter principles have never been translated into coating applications. Drying coatings provide a dynamically evolving landscape, with moving interfaces, increasing confinement, and changing physicochemical conditions, making them a particularly relevant platform to explore active matter behavior beyond equilibrium settings.

In this work, we report for the first time the introduction of active swimmers into coating formulations. We create coatings from blends of active JCs and passive film‐forming particles by exploiting the competition between active particle fuel depletion and water evaporation. We find that active JCs self‐propel and accumulate at interfaces in the system, i.e., air/water and substrate/water. This results in active particle enrichment at both the top and bottom of the coatings. Furthermore, we examine the orientation of active JCs in the dried coatings and find an orientational bias at the film surface. Our results show the potential for active JCs as a tool to control coating microstructure, and pave the way for future work to use active colloids to drive functionality where desired. In our study, we employ $H_{2}$ $O_{2}$ fuelled Pt‐coated JCs as a model system, but the underlying concept is not restricted to this specific propulsion mechanism. The key requirement is not the chemistry of propulsion, but rather the ability to sustain particle activity over timescales comparable to those of film formation. Therefore, the introduction of active ingredients based on other actuation strategies such as light‐induced propulsion, thermophoretic motion, magnetic actuation, or enzyme‐powered systems would be expected to follow similar trends.

## 2 Results and Discussion

### 2.1 Balancing Evaporation‐Driven Film Formation with Fuel Depletion

**FIGURE 1:**
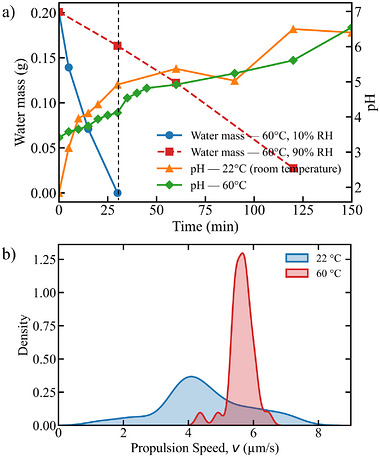
(a) pH evolution over time for active JC suspension at room temperature ( 22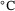, orange triangles) and 60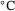 (green squares). Variation of water mass during film formation at 60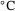, 10% RH (blue circles) and 60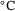, 90% RH (red squares). (b) Propulsion speed distribution of active JCs at room temperature (22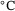) and 60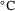. Higher temperature leads to faster and more consistent particle propulsion speeds.

Platinum coated polystyrene (*Pt*‐PS) JCs (2.00±0.02μm in diameter) were prepared as described in the Supporting Information, and dispersed in deionized water via sonication. pH measurements of the suspension containing JCs, with 10% hydrogen peroxide added, were recorded as a proxy to identify the time point of fuel depletion at room temperature and 60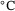. The catalytic decomposition of $H_{2}$ $O_{2}$ on platinum surfaces leads to the formation of reactive intermediates and ionic species which raise the pH of the suspension. At acidic pH, platinum can promote the formation of hydroxyl radicals or hydroxide ions at its surface rather than proceeding via a fully neutral decomposition pathway [36]. Over time, the pH of our JCs dispersions is thus expected to increase and eventually reach a plateau when all the $H_{2}$ $O_{2}$ is consumed, enabling us to determine the time for fuel depletion. As shown in Figure 1a (orange triangles and green squares), the pH of the dispersion increases rapidly over 30 min and starts to reach a plateau thereafter. The final pH values resemble those reported for pure water exposed to atmospheric ${\mathrm{CO}}_{2}$. Importantly, the cessation of particle motion was consistently observed after approximately 30 min (see Video S1), proving the suitability of pH as a proxy to detect fuel depletion in our system. The observed hydrogen peroxide depletion rates suggest that, if JCs were introduced into coatings formulations dried under room temperature conditions ( 22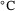 and ∼50% relative humidity, RH), catalytic activity would be lost long before the film structure is fully developed, as this typically occurs over several hours [14]. Therefore, to accelerate the water evaporation rate and ensure the film structure is set before hydrogen peroxide is depleted, we prepared our films at 60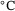. At this temperature and 10% RH all the water is evaporated before fuel depletion occurs (see Figure 1a, blue circles), ensuring the activity of the JCs plays a role in the development of the coating microstructure. We chose a second environmental condition, 60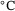 and 90 % RH (red squares), as a control for a scenario when the activity of the particles is lost before the film structure is fully set (Figure 1a, red squares).

We then investigated the dynamics of active JPs under these high temperature conditions, using a microscope equipped with a temperature‐controlled stage. In our system, propulsion is driven by the exothermic decomposition of hydrogen peroxide, whose rate increases with temperature according to the Arrhenius equation [21, 37]. The observed distributions (Figure 1b) clearly demonstrate that the propulsion speeds of active JCs significantly increase at 60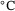 when compared to those measured at room temperature. Higher temperatures accelerate the reaction kinetics, leading to steeper chemical gradients and, in turn, stronger self‐diffusiophoretic propulsion forces on the active JCs [21, 38]. Interestingly, active JCs not only exhibit faster propulsion speeds but also a narrower distribution. Although a detailed investigation of this observation lies beyond the scope of the present work, we are not aware of prior reports of this phenomenon.

To summarize the competition between relevant physical processes, we compile the characteristic timescales governing these active systems in Table 1. They include the active lifetime of the Janus colloids, the water evaporation time under different environmental conditions, and the JCs sedimentation time. As we will prove later in this work, balancing these timescales is key to achieve control over the microstructure of coatings containing active JCs in their formulation.

**TABLE 1:** Characteristic timescales governing the JCs system.

Process	Timescale	Notes
Activity lifetime (fuel depletion)	∼ 30 min	From pH plateau and cessation of particle motion
Water evaporation (60 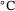 , 10% RH)	∼ 30 min	Film structure is set before fuel depletion
Water evaporation (60 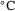 , 90% RH)	> 2 h	Fuel depleted before film structure is set
Sedimentation time	≫ 30 min	Based on settling velocity ($∼0.11\;\mu m\;s^{-1}$)

### 2.2 Film Formation and Microstructure

**FIGURE 2:**
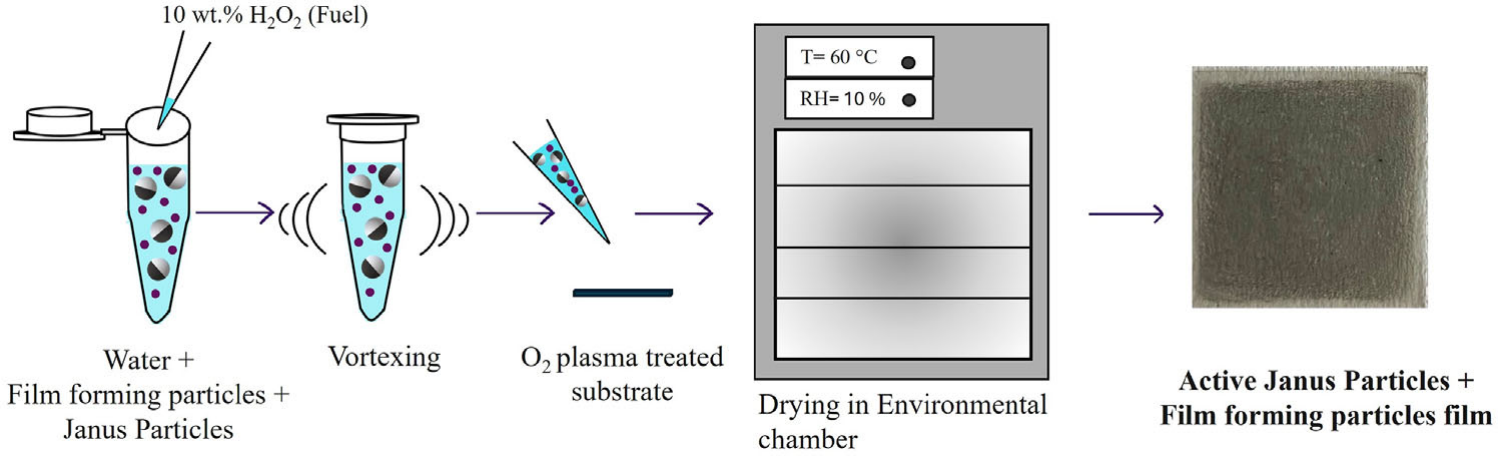
Experimental setup used for coatings formulation and film formation.

**FIGURE 3:**
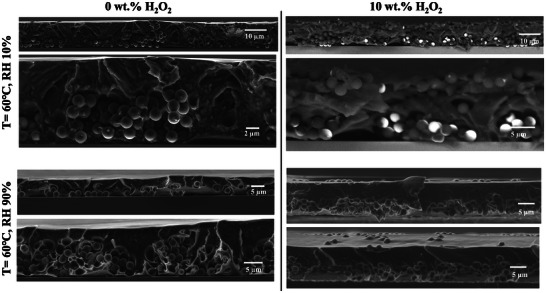
Cross‐section SEM images of coatings prepared from formulations without (left column) and with (right column) active JC fuel, film formed either at 60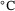 and 10% RH (top images) or 60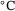 and 90% RH (bottom images). In the absence of fuel or at slower evaporation rates, JCs predominantly sediment to the bottom of the film. In the presence of fuel and at faster evaporation rates, active JCs accumulate both at the top and bottom interfaces due to their self‐propulsion.

Coating formulations were prepared by blending a film‐forming polymer (binder) particle dispersion (diameter of 224±0.02nm, synthesis described in the Supporting Information), a JCs dispersion, deionized water, and hydrogen peroxide to obtain a final dispersion containing 0.3wt.% active JCs, 3wt.% binder particles, and 10 wt.% H_2_O_2_ (Figure 2). Control formulations without H_2_O_2_ were prepared to serve as a baseline for dispersions with only Brownian motion. 200μL of these dispersions were cast onto plasmatreated (HPT‐100, Henniker Plasma) glass coverslips ($18\times 18\;{\mathrm{mm}}^{2}$). The formulations were then placed in an environmental chamber under controlled temperature and relative humidity to film form. Two film formation regimes were implemented, as described earlier: fast‐drying ($T=60^{\circ }C$, RH = 10%) and slow‐drying ($T=60^{\circ }C$, RH = 90%). Under fast‐drying conditions, water evaporation was typically complete within 30 min, whereas under slow‐drying conditions, drying required in excess of 2 h (see Figure 1a). Figure 3 presents scanning electron microscopy (SEM) micrographs of cross‐sections of the resulting films, revealing the JCs distribution within the coatings. Binder particles lose their spherical shape because their glass transition temperature ($T_{g}$) is well below the temperature used for film formation (see Figure S1). After water evaporates, binder particles deform, coalesce, and their chains interdiffuse across boundaries [39] to create a matrix that supports the JCs. If no H_2_O_2_ is added to the formulation, regardless of using slower or faster evaporation rates the JCs are always found close to the substrate at the bottom of the coatings (Figure 3, left column). In these scenarios, both JCs and binder particles undergo Brownian motion only. In the absence of fuel, the JCs cannot self‐propel and are therefore subject to sedimentation due to their large diameter [40].

However, by introducing fuel used for self propulsion of JCs and carefully balancing its depletion timescale with that of water evaporation, we obtain a strikingly different coating microstructure. Formulations containing H_2_O_2_ formed at 60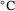 and 10% RH result in films with both the top and bottom surfaces enriched in JCs (Figure 3, right column, top). Such vertical distribution highlights the ability of the active JCs to overcome gravity during film formation in the presence of H_2_O_2_. Their self‐diffusiophoretic motion enables them to transport to one of the two main interfaces (air/water or substrate/water) and remain there [41, 42]. The self‐propulsion speed of active JCs at 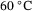 and 10% RH (4−6μm/s) overwhelmingly exceeds their settling velocity (0.11±0.01μm/s) as well as the air/water interface recession speed (0.41±0.20μm/s). Importantly, when the water evaporation rate is decreased by increasing RH to 90%, the JCs are found only at the bottom of the coatings. In this system, H_2_O_2_ is depleted well before the film structure settles (Figure 1a). This confirms that, under purely Brownian motion, JCs sediment to the bottom, and that the sandwich‐like coating structure formed by casting formulations with $H_{2}O_{2}$ at 60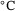 and 10% RH arises from their self‐propulsion.

**FIGURE 4:**
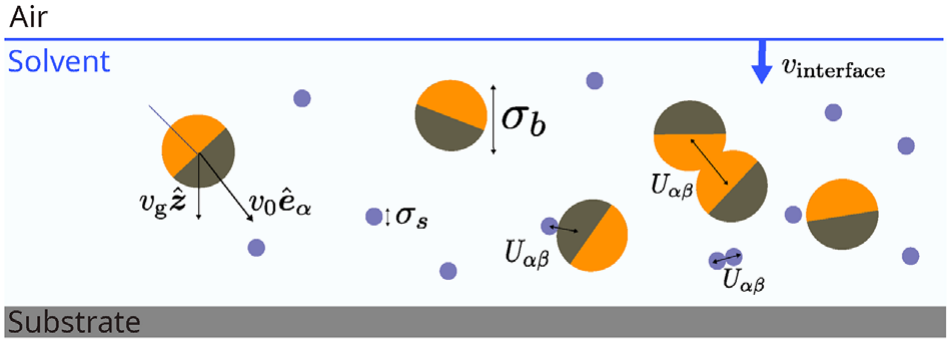
Schematic depiction of the simulated binary mixture of particles of diameters $\sigma_{b}$ and $\sigma_{s}$. Large particles represent active JCs self‐propelling with speed $v_{0}$ and sedimenting due to gravity with speed $v_{g}$. Particle pairs α,β interact through an interaction potential $U_{\alpha \beta }$. The air‐water interface moves at a constant speed $v_{\text{interface.}}$

To gain insights into the early stages of drying and better understand the underlying mechanisms, we performed numerical simulations of a simplified model system schematically depicted in Figure 4. Given the relevant lengths and velocities, we assume a vanishing Reynolds number and perform simulations of an overdamped active Brownian particle (ABP) dynamics. The first component of the mixture is small, neutrally buoyant Brownian particles of diameter $\sigma_{s}$. Their positions $x_{s,i}$ are described by an equation of motion

(1) $$\begin{array}{cc} \frac{dx_{s,\alpha }}{dt} & =-\gamma_{s}^{-1}\nabla U+\sqrt{2D_{s}}\xi_{s,\alpha }\left(t\right) \end{array}$$

The second component is larger, sedimenting active Brownian particles [18, 43, 44] of diameter $\sigma_{b}$ described by their positions $x_{b,\alpha }$ and a unit vector ${\hat{e}}_{\alpha }$ in the direction of propulsion

(2) $$\begin{array}{cc} \frac{dx_{b,\alpha }}{dt} & =v_{0}{\hat{e}}_{\alpha }-v_{g}\hat{z}--\gamma_{b}^{-1}\nabla U+\sqrt{2D_{b}}\xi_{b,\alpha }\left(t\right) \end{array}$$

(3) $$\begin{array}{cc} \frac{d{\hat{e}}_{\alpha }}{dt} & =\sqrt{2D_{R}}\zeta_{\alpha }\left(t\right)\times {\hat{e}}_{\alpha } \end{array}$$

We use a Greek letter subscript to differentiate different individual particles and the subscripts s/b to differentiate small and big particles. Translational and rotational diffusion constants are given by the Stokes‐Einstein relations $D_{\text{s/b}}=k_{B}T/\left(3\pi \mu \sigma_{\text{s/b}}\right)$ and $D_{R}=k_{B}T/\left(\pi \mu \sigma_{b}^{3}\right)$, respectively, and drag constants $\gamma_{\text{s/b}}=3\pi \mu \sigma_{\text{s/b}}$. ABPs move with a constant self‐propulsion velocity $v_{0}$ in the direction of their orientation and sediment at a constant speed $v_{g}$ in the z‐direction. The potential U describes the pairwise particle interactions and their interactions with interfaces through a short range repulsive potential. We keep the bottom interface fixed at z=0 and move the top interface downwards with constant speed $h\left(t\right)=h_{0}-v_{\text{interface}}\tilde{t}$ to simulate a constant evaporation rate. The components of the random vectors $\xi_{\text{s/b},\alpha }$ and $\zeta_{\alpha }$ are sampled from delta‐correlated Gaussian distributions with unit width such that $\left\langle \xi_{\alpha ,i}\left(t\right)\xi_{\beta ,j}\left(t^{\prime }\right)\right\rangle =\left\langle \zeta_{\alpha ,i}\left(t\right)\zeta_{\beta ,j}\left(t^{\prime }\right)\right\rangle =\delta_{\alpha \beta }\delta_{ij}\delta \left(t-t^{\prime }\right)$.

Nondimensionalizing Equations (1)–(3) such that $\tilde{x}=x/\sigma_{b}$ and $\tilde{t}=tD_{b}/\sigma_{b}^{2}$ yields equations of motion

(4) $$\begin{array}{cc} \frac{d{\tilde{x}}_{s,\alpha }}{d\tilde{t}} & =-\tilde{\sigma }\tilde{\nabla }\tilde{U}+\sqrt{2\tilde{\sigma }}{\tilde{\xi }}_{s,i}\left(\tilde{t}\right) \end{array}$$

(5) $$\begin{array}{cc} \frac{d{\tilde{x}}_{b,\alpha }}{d\tilde{t}} & ={\mathrm{Pe}}_{\text{active}}{\hat{e}}_{\alpha }-{\tilde{v}}_{g}\hat{z}-\tilde{\nabla }\tilde{U}+\sqrt{2}{\tilde{\xi }}_{b,\alpha }\left(\tilde{t}\right) \end{array}$$

(6) $$\begin{array}{cc} \frac{d{\hat{e}}_{b,\alpha }}{d\tilde{t}} & =\sqrt{6}{\tilde{\zeta }}_{\alpha }\times {\hat{e}}_{\alpha } \end{array}$$

The dimensionless equivalents to parameters in Equations (1)–(3) are marked with a tilde. Further details on the nondimensionalization can be found in the Supporting Information. We introduce another Péclet number that compares the advective effects of active self‐propulsion with the strength of diffusion ${\mathrm{Pe}}_{\text{acitve}}=v_{0}\sigma_{b}/D_{b}$ and a size ratio $\tilde{\sigma }=\sigma_{b}/\sigma_{s}$.

We stop our simulations once the distance between top and bottom interfaces corresponds to ten times the diameter of the large particles, defining $t_{\text{final}}\equiv \left(h_{0}-10\right)/{\tilde{v}}_{\text{interface}}$, where $h_{0}$ is the initial box height. At this stage, the system is jammed, and the film microstructure is locked in place. It is important to acknowledge that this model neglects a number of the complexities present in the experimental system, such as hydrodynamic interactions, adsorption effects, or the consumption of hydrogen peroxide fuel over time. These limitations prevent us from making a direct quantitative comparison with experiments, but we are still able to qualitatively capture the key phenomena, i.e., the competition between evaporation‐driven assembly and active particle propulsion, and how this is sufficient to drive the observed microstructure and the polarization of active particles at interfaces.

**FIGURE 5:**
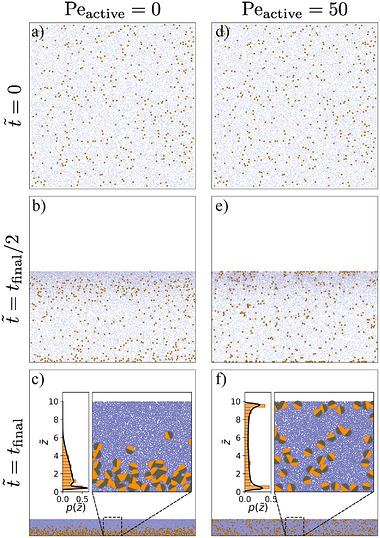
Time evolution of the simulated system for two blends: small passive colloids with large passive colloids (left) and small passive colloids with large active colloids (right). A zoom into the final film configuration is shown as an inset. The accompanying histograms show the vertical distribution of JCs averaged over five repeated simulations. To estimate the underlying distribution shown with a black line, a kernel density estimation (KDE) method is used that respects the boundary conditions (see Supporting Information for a detailed description). In the passive–passive case ($Pe_{\text{active}}=0$) all JCs are located at the bottom of the film. In the passive‐active case ($Pe_{\text{active}}=50$) the distribution function has two distinct peaks at the top and at the bottom of the final film.

The time evolution of the two simulated systems is shown in Figure 5. In a blend of small passive and large passive particles, as drying proceeds small particles are swept by the moving interface. This results in a chemical gradient which drives large particles downwards [10, 12]. This downward trend is observed even in the absence of gravitational effects (simulations without gravity are shown in the Supporting Information), but it becomes more pronounced when the buoyancy of the JCs, as present in the experiments, is taken into account. A region dense in small particles is detected at very early drying stages (see Figure 5b), and it leads to an upper region of the film at $t_{\text{final}}$ heavily enriched in them (see Figure 5c). Our observations on the evaporation‐driven assembly of passive–passive colloidal mixtures are in good alignment with previous studies [9, 10, 11, 12].

**FIGURE 6:**
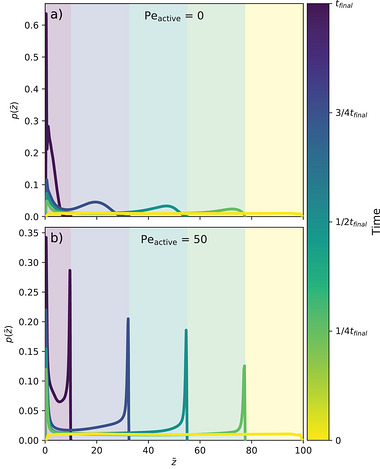
Normalized probability density distributions of the active particles as a function of height $\tilde{z}$ at different times for (a) passive–passive, and (b) passive‐active mixtures. Each curve represents the KDE estimate of the particle height distribution at given stage of the drying process. Distributions are obtained by averaging over five independent simulation repetitions for each activity level. Different times in the drying process are denoted by color, with the background showing the corresponding film height.

As we observed in our experiments, simulations of passive‐active blends show that when big particles are able to self‐propel, they not only overcome the pull of gravity [45, 46, 47], but also the gradient in chemical potential generated by the dense region of small particles near the moving interface [10, 11, 12]. As a consequence of the wall‐hugging properties of spherical active particles [18, 48, 49, 50, 51, 52] they accumulate at both the water/air and water/substrate interfaces (see Figure 5e). As the air/water interface moves downwards, the system is compressed, increasing the small particle density and reducing particle mobility until the system ultimately jams (see Figure 5f). While passive colloids gather exclusively at the bottom interface (see Figure 6a), the vertical density distribution $p(\tilde{z})$ of the active particles is highly bimodal with sharp peaks at the bottom and top interface with only a slight excess of particles at the bottom (see Figure 6b). In contrast to the simulations of passive–passive blends, a significant fraction of the active particles is found at the top interface in agreement with the experimental SEM observations in Figure 3.

**FIGURE 7:**
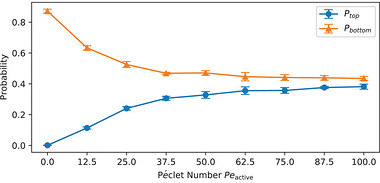
Fraction of big particles found in the upper and lower third of the film at height 10. Circles correspond to the top fraction and triangles to the bottom fraction, averaged over five repetitions for each simulation. Without activity (${\mathrm{Pe}}_{\text{active}}=0$) the absolute majority of the active particles sink at the bottom. Activity is positively correlated with the number of particles at the top, and negatively correlated with the number of particles at the bottom.

To investigate the dependence of active particle distribution with their degree of activity, we calculate the probabilities of finding them at the bottom $P_{\text{bottom}}=\int_{0}^{w}p\left(\tilde{z}\right)d\tilde{z}$ or the top of the final film $P_{\text{top}}=\int_{h(t_{\text{final}})-w}^{h(t_{\text{final}})}p\left(\tilde{z}\right)d\tilde{z}$. To capture the full width of the density peaks at the bottom and top of the film, we choose $w=h(t_{\text{final}})/3$. Figure 7 shows that when particles are not active (${\mathrm{Pe}}_{\text{active}}=0$) almost all large particles are found near the bottom. With increasing activity, more large particles escape the bottom interface and accumulate at the top interface. At high activity, chemical potential gradients and gravity do not seem to play a role in distinguishing the top and bottom interfaces, and large particles are split evenly between the two with only a small fraction of ∼20% remaining in the middle of the film. To confirm the robustness of our results, additional simulations were performed varying the pair potential used, the influence of relative particle size, and the relative density of big and small particles (see Supporting Information). We universally observe the relative size difference of particles to lead to small‐on‐top stratification for passive–passive blends, which is overcome with sufficient activity to lead to a sandwich‐like structure.

### 2.3 Surface Coverage and Janus Orientation

**FIGURE 8:**
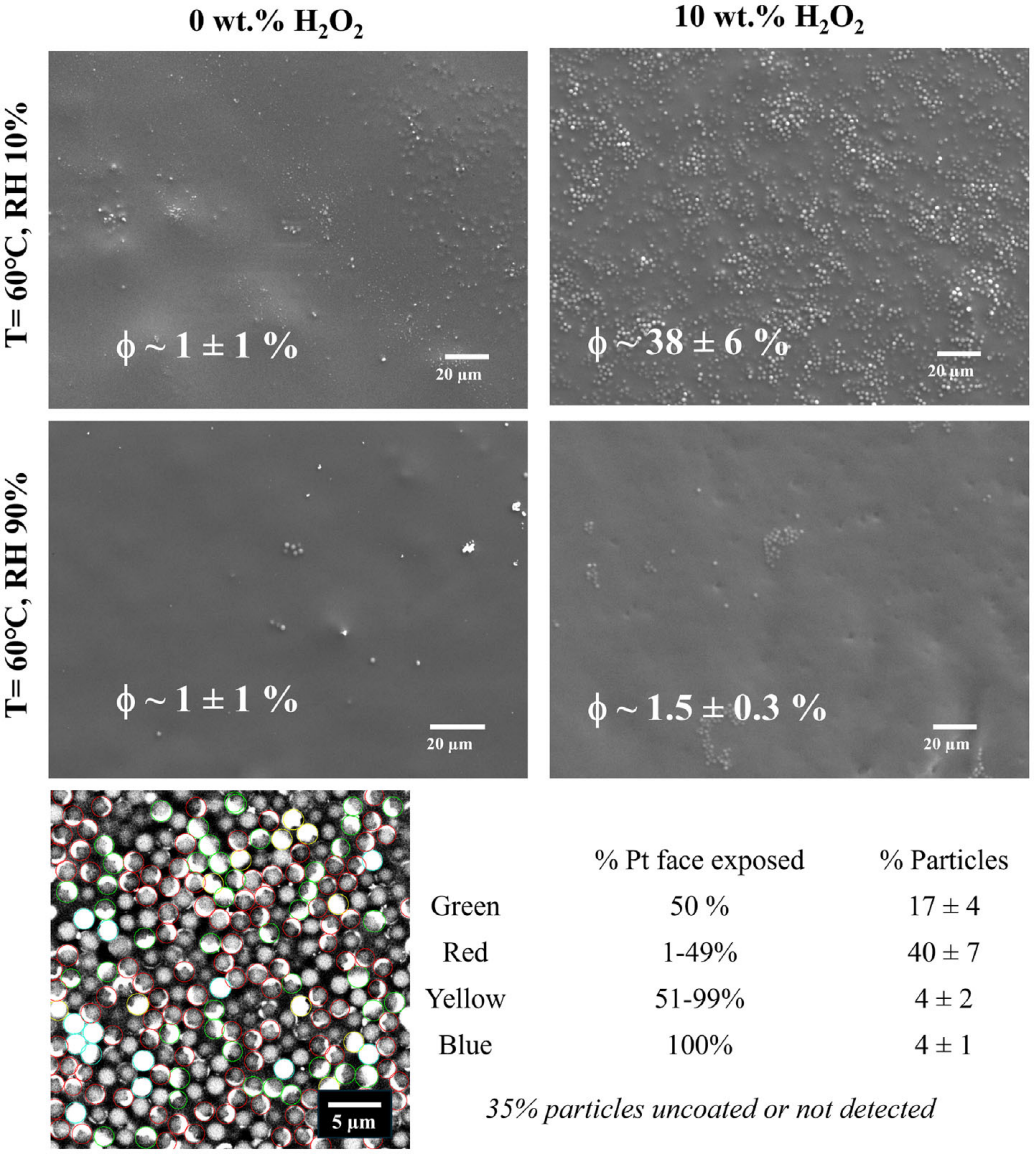
Top‐view SEM images of the upper surface of films dried under different environmental conditions, without and with active JC fuel in the formulation. Zoomed in image shows the surface coverage of the active JCs at $T=60^{\circ }C$ and RH=10%.

As well as the vertical particle distribution across the film thickness, from a functional coatings point of view, it is important to investigate the surface coverage by JCs at the top surface. As shown in Figure 8, the fraction of the surface area occupied by JCs (ϕ) is strongly influenced by both environmental conditions and the presence or absence of active JC fuel. The parameter ϕ was quantified by image analysis of multiple SEM micrographs for each condition. In coatings formed at $T=60^{\circ }C$ and RH=10%, only sparse particles at the top surfaces are observed in the absence of hydrogen peroxide (ϕ∼1±1%). In contrast, when $H_{2}$ $O_{2}$ is introduced in the formulation, a significantly higher surface coverage is observed (ϕ=38±6%) under the same environmental conditions. Under slow drying conditions ($T=60^{\circ }C,\text{RH}=90\%$), the surface coverage by JCs remains low irrespective of the presence of fuel, with measured values of ϕ∼1±1% (no fuel) and ϕ∼1.5±0.3% (with fuel). These results confirm, at a larger scale, the features observed in the cross‐sectional SEM images.

The use of backscattered electron (BSE) images, as shown in Figure S2, enables the investigation of JCs orientation in passive‐active formulations. By means of image analysis, JCs located at the top of the coatings can be classified according to the visible fraction of their *Pt*‐coated face oriented toward the air phase (Figure 8). Approximately 17±4% of particles displayed an equatorial orientation (50% *Pt* face visible, green), 40±7% sub‐equatorial orientation (1−49% *Pt* face visible, red), 4±2% supra‐equatorial orientation (51−99% *Pt* face visible, yellow), and 4±1% were cap‐up (100% *Pt* face visible, blue). A considerable fraction, approximately 35%, either appeared uncoated or could not be reliably identified due to partial coalescence of the film‐forming matrix around the particles (see Figure S3 and associated text in Supporting Information). These particles were included in the overall count but grouped within the non‐resolved fraction whenever their orientation could not be determined unambiguously. This analysis confirms that, while a significant number of JCs accumulate at the top surface during fast drying with fuel, their orientations are heterogeneous. The combination of surface coverage ϕ and *Pt* face orientation statistics provides a comprehensive picture of the coating surface.

**FIGURE 9:**
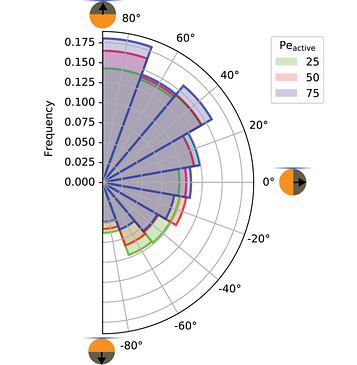
Distribution of active particle orientations $\theta =\arcsin(e_{\alpha ,z})$ in the upper third of the film at height $t_{\text{final}}$. Active particles show a bias of orientations toward the boundary (θ>0) increasing with Péclet number ${\mathrm{Pe}}_{\text{active}}$. Note that this analysis does not differentiate particles moving left or right, given the symmetry of the system in the x‐direction.

The propensity of active particles to accumulate near boundaries results in an orientational bias in the vicinity of interfaces. An active particle oriented toward an interface will swim toward it and remain at the interface colliding with it, until particle orientation changes enough through rotational diffusion to be directed away from the interface. This results in a typical wall hugging time $\propto D_{R}^{-1}$ [48, 49, 51, 52, 53]. Once its orientation ${\hat{e}}_{\alpha }$ no longer points to the interface, the active particle will quickly escape the interface via self‐propulsion. We investigate this effect by analyzing the angle θ between the active particle orientations ${\hat{e}}_{\alpha }$ in the upper third of the final film produced by our simulations, as presented in Figure 9. In line with the argument presented above, near the top interface we find an excess of particles pointing towards the interface $\theta >0^{\circ }$ compared to particles pointing away from it $\theta <0^{\circ }$‐ as we observed in our experiments. The strength of this effect increases with increasing Péclet number. Faster particles make their way towards the interface quicker when oriented toward it, and are also able to escape quicker once they change their orientation. This effect presents a possible explanation for the observed bias of JCs in experiments to present more of their uncoated side at the top of a dried film, as shown in Figure 8.

## 3 Conclusions

Our work successfully incorporates, for the first time, active matter principles into coatings. We presented a proof‐of‐concept study which proves that active JCs can be incorporated into coatings formulations to influence microstructure during the film formation process. We have moved beyond passive dynamics and assembly, demonstrating a novel, non‐equilibrium pathway to dictate the spatial distribution of formulation ingredients within coatings.

Working with formulations composed of active JCs and film‐forming particles, we learned that the capacity for microstructure control relies on establishing a critical balance between two competing timescales. The first is the characteristic time for the evaporation‐induced assembly, and the second is the time required for active particle fuel (i.e., hydrogen peroxide) depletion. We reveal that this kinetic interplay is the decisive factor governing the period of active migration, allowing for tunability of coating microstructure.

Leveraging this mechanism, we demonstrated the ability to program the final distribution of active JCs, showing their directed accumulation at both the air‐coating interface (top surface) and the coating‐substrate interface (bottom surface). In the presence of fuel, active JCs are able to overcome both sedimentation and chemical potential gradients induced by moving interfaces and anisotropy in film‐forming particle distributions. This observation is supported by numerical simulations of a simplified model system, which qualitatively captures the key phenomena occurring during the film formation process and leads to similar coating microstructures as those observed in experiments.

JCs showed a heterogeneous orientation at the top interface, with the sub‐equatorial orientation being the most common, resulting in the JCs overall presenting more of their uncoated side at the top surface. The observed bias can be explained by the polarization active particles show near boundaries  [52, 54], and confirmed by our modelling results. This bias is predicted to be more pronounced when the colloid activity increases, thus providing a mechanism to control particle orientation at the air‐coating interface.

This study not only presents the first successful incorporation of self‐propelled, active components into coatings formulations, but also can pave the way for future advanced functional coatings with programmable ingredient distribution. It is important to note that while the present study employs Pt‐coated JCs fuelled by $H_{2}$ $O_{2}$ as a model system, the underlying concept is not restricted to this specific propulsion mechanism. Therefore, alternative actuation strategies, including light‐driven [55], thermophoretic [56], magnetic [57], and enzyme‐powered systems [58], could also be harnessed. There are many examples where functional ingredients are desirable at both the top and bottom of a coating, especially when it comes to corrosion and barrier (moisture or gas) protection. Moreover, the use of JCs and further optimization of their orientation at interfaces could enable surfaces with dual functionality, each of them determined by one of the JC faces.

Future work should address the gap between the proof‐of‐concept presented here and the industrial application. This includes testing the influence of hydrogen peroxide on the binder and final film properties (e.g., adhesion), as well as on the stability of additives and the overall system in a full formulation. In addition, further research is needed to identify viable strategies for scaling up the production of Janus particles, which are currently limited to laboratory‐scale synthesis.

## Author Contributions

K.S.: Conceptualization, Methodology, Data collection, Curation and Analysis, Validation, Visualization, Writing (Original draft, Review and Editing). J.C. (the author not the colloids): Conceptualization, Simulations, Writing (Original draft ‐ Simulations, Review and Editing). L.B.: Simulations, Visualization. E.E.R.: Resources (binder particles), Writing (Original draft ‐ binder synthesis, Review and Editing). F.D.: Resources (binder particles), Writing (Original draft ‐ binder synthesis, Review and Editing). M.L.: Resources (binder particles), Writing (Original draft ‐ binder synthesis, Review and Editing). I.M.F.: Conceptualization, Supervision, Resources, and Writing (Review and Editing). All authors have read and approved the final manuscript.

## Conflicts of Interest

The authors declare no conflicts of interest.

## Supporting information


**Supporting File 1**: smll73903‐sup‐0001‐SuppMat.pdf.

**Supporting File 2**: smll73903‐sup‐0002‐VideoS1.avi.

## Data Availability

The data that support the findings of this study are available from the corresponding author upon reasonable request.
