# Supplementary material for: From Passive to Active: Self‐Propelled Colloids in Coatings Formulation and Film Formation
Source: Small. 2026 May 31;22(39):e73903. doi: 10.1002/smll.73903 (PMC13360545; doi:10.1002/smll.73903)
Supplement: Supplementary file 1 — Supporting File 1: smll73903‐sup‐0001‐SuppMat.pdf. [file SMLL-22-e73903-s002.pdf]

From Passive to Active: Self-Propelled Colloids in Coatings Formulation and Film Formation

Karnika Singh,[1,*] Jan Cammann,[2,*] Luka Burduli,[2,3] Edgar Espinosa Rodriguez,[4] Franck D'Agosto,[4] Muriel Lansalot,[4] and Ignacio Martin-Fabiani[1,†]

[1] *Department of Materials, Loughborough University, Loughborough LE11 3TU, United Kingdom*
[2] *Interdisciplinary Centre for Mathematical Modelling and Department of Mathematical Sciences, Loughborough University, Loughborough LE11 3TU, United Kingdom*
[3] *School of Science, Constructor University, Campus Ring 1, 28759 Bremen, Germany*
[4] *Catalysis, Polymerization, Processes and Materials (CP2M), Universite Claude Bernard Lyon 1, CPE Lyon, CNRS, UMR 5128, Villeurbanne F-69616, France*

(Dated: May 1, 2026)

SUPPLEMENTARY INFORMATION

I. MATERIALS AND METHODS

A. Active Janus Particles Preparation

Isotropic, spherical polystyrene (PS) microparticles with a diameter of $2.00 \pm 0.02\,\mu$m were purchased from Thermo Fisher Scientific (10 wt.%). This 10 wt.% PS aqueous dispersion was mixed with ethanol to reduce the solids content to 2 wt.% and cast onto a plasma cleaned glass slide held at 50°C on a temperature-controlled hot plate. The elevated substrate temperature, in combination with the high volatility of ethanol, promotes uniform, rapid evaporation with minimal contact line pinning. This enables the formation of a close-packed monolayer of PS particles, as verified by optical microscopy.

Then, a platinum coating of thickness ~ 14 nm was deposited on the PS particle monolayer by DC magnetron sputtering using a benchtop sputter coater (Q150T S, Quorum). Owing to the self-shadowing effect inherent to line-of-sight deposition on a close-packed bed, only the exposed hemispheres were coated, thereby rendering the particles Janus (*Pt*-PS).

B. Film-forming particles synthesis

Reversible addition-fragmentation chain transfer (RAFT) polymerization was used to synthesize water soluble polymers (macroRAFT agents) further employed in the production of film-forming passive particles. The protocol used for the synthesis of the macroRAFT agent was adapted from the experimental conditions described in Velasquez *et al.* [1]. First, 200 g of deionised water was added to a 500 mL double-jacket glass reactor equipped with a condenser. Then, 4-cyano-4-thiothiopropylsulfanylpentanoic acid (CTPPA) (1.7 g, 6.1 mmol), 4-styrene sulfonate (SSNa) (19 g, 92 mmol) and sodium bicarbonate ($NaHCO_3$) (0.15 g, 1.8 mmol) were added to the reactor as well. The mixture was deoxygenated with nitrogen for 30 minutes and then heated to 80°C, and the stirring was set at 250 rpm. To start the synthesis, 4,4-azobis(4-cyanopentanoic acid (ACPA) (0.15 g, 0.6 mmol) was added together with 1,3,5-trioxane (3.3 g, added as an internal reference for NMR analyses) in 1 mL of deionised and deoxygenated water. Full conversion analysis (determined by ^{1}H NMR) was achieved after 3 hours. The recovered solution had a solids content of 8.5 wt.%. A sample was dried and the polymer recovered was analysed by Matrix-Assisted Laser Desorption/Ionization – Time-Of-Flight (MALDI-ToF) mass spectrometry to determine the molar mass of the macroRAFT agent: M_n(MALDI-ToF) $= 3021\,\mathrm{g\,mol^{-1}}$.

Emulsion copolymerizations of n-butyl acrylate (BA) and methyl methacrylate (MMA) (50/50 % in wt.) were performed at 70°C in a 500 mL double-jacket glass reactor equipped with a condenser using the PSSNa macroRAFT agent and targeting a final solids content close to 20 wt.%. 150 g of deionised water were poured into the reactor and deoxygenated for 30 minutes. BA (20 g, 0.20 mol) and MMA (20 g, 0.16 mol) were then added to the reactor, followed by 3.54 mL of the macroRAFT solution (0.26 g, 0.086 mmol). The mixture was stirred at 250 rpm and heated to 70°C. 0.07 g of ammonium persulfate (APS) (0.3 mmol) dissolved in 1 mL of deionised and deoxygenated water was added to start the polymerization. The reaction was stopped after 4 hours when full conversion was achieved, as determined by gravimetric analysis. The final film forming particles had a 21.2 wt.% solids content.

TABLE S1. Physicochemical properties of the particles used in the study.

Particle Type	Diameter, d	Zeta Potential, ζ (mV)
Polystyrene particles	$2.00 \pm 0.02\,\mu$m	-28 ± 2
Film-forming particles	224 ± 1 nm	-50 ± 1

* equal contribution
† I.Martin-fabiani@lboro.ac.uk

C. Dynamic and electrophoretic light scattering

Particle diameter and surface charge (ζ-potential) were measured using a Zetasizer Ultra (Malvern Panalytical, Malvern, U.K.). All measurements were conducted at 25°C using folded capillary cells (DTS1070). Each sample was measured five times to ensure reproducibility. Polystyrene and film forming particle suspensions were diluted to 0.1 wt.% prior to measurement. Both types of particles were selected to be negatively charged in order to minimize aggregation arising from electrostatic interactions.

D. Measurement of water evaporation rates

A 200 μL droplet of deionized water was deposited onto a coverslip pre-treated with oxygen plasma to render the surface hydrophilic and ensure uniform spreading. Then, it was placed inside an environmental chamber (HPP IPP plus, Memmert, Germany) maintained at $T = 60$°C and RH 10% or 90 %, for drying - depending on whether we wanted to measure evaporation rate for fast or slow drying conditions. At RH = 10%, the water mass was recorded every 5 minutes whereas at RH = 90%, mass loss was measured at 30-minute intervals. To avoid interference on the measurements from temperature/RH stabilisation when opening and closing the chamber, a fresh water sample was used for each measurement.

E. Sample preparation for pH studies

A suspension of Janus colloids (JCs) was prepared at the same particle weight fraction used for film formation. 10 wt% hydrogen peroxide was added to the suspension. Measurements were conducted at both room temperature ($T = 22$°C) and at 60 C on a hot plate. In both cases, the pH of the suspension was monitored for a period of 150 minutes using a Thermo Scientific™ Orion Star™ A211 Benchtop pH Meter.

F. Differential scanning calorimetry

DSC measurements were carried out using a *TA Instruments Q200* calorimeter to determine the glass transition temperature (T_g) of the samples. Approximately 5–10 mg of each sample was sealed in an aluminum pan and subjected to a controlled thermal cycle under a constant flow of nitrogen gas to prevent oxidative degradation.

G. Imaging and tracking

Isolated active particle motion was recorded by means of optical microscopy using a Keyence VHX7000N digital microscope on a temperature-controlled stage (Mettler Toledo FP90 Hotstage). Samples were equilibrated at the target temperature before acquisition, and videos were captured at 20 fps.

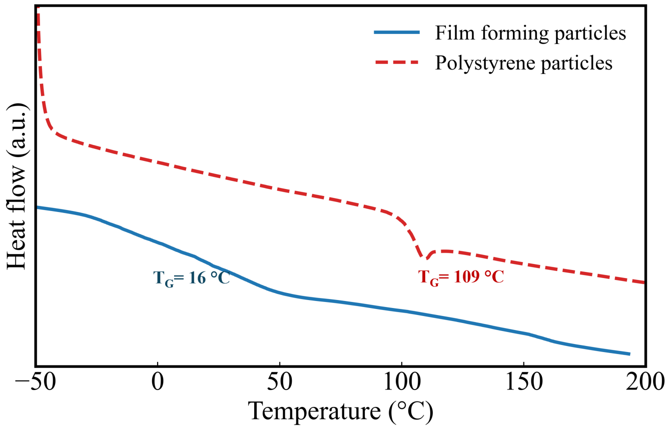

FIG. S1. DSC thermograms of film forming particles and polystyrene particles. The film forming particles exhibit a Tg of 16°C, while the polystyrene particles show a Tg of 109°C

Trajectories were extracted in *Fiji*/ImageJ using the TrackMate plugin. A single set of detection, linking, and filtering settings was established on a representative dataset and applied unchanged to all temperatures to ensure consistency.

Instantaneous speeds were computed as

$$v_i(t_k) = \frac{\|\mathbf{r}_i(t_{k+1}) - \mathbf{r}_i(t_k)\|}{\Delta t}, \qquad \Delta t = \frac{1}{20}\ \mathrm{s}.$$

For each temperature, speed statistics (distributions and median values) were compiled across multiple fields of view, using the same workflow for all datasets.

H. Scanning electron microscopy (SEM)

A thin carbon coating was applied then to improve surface conductivity. SEM imaging was conducted using a JEOL JSM-7100F field emission gun scanning electron microscope (FEG-SEM) at an accelerating voltage of 5 keV to minimize beam damage. A thin carbon coating was applied to the sample to improve surface conductivity. To image cross-sections of our coatings, some samples were fractured into smaller pieces by applying pressure at their center with a diamond scriber.

To study the JC orientation, we combined backscattered electron (BSE) imaging (at 10 keV) together with energy-dispersive X-ray spectroscopy (EDS) mapping (Oxford Instruments X-Max 80 mm^2 detector). Figure S2 shows how the higher contrast areas observed in the BSE images correspond to regions of the JCs coated by platinum, enabling us to quantify and study their orientation. In addition to identifying the platinum cap, the BSE micrographs also reveal the presence of a continuous

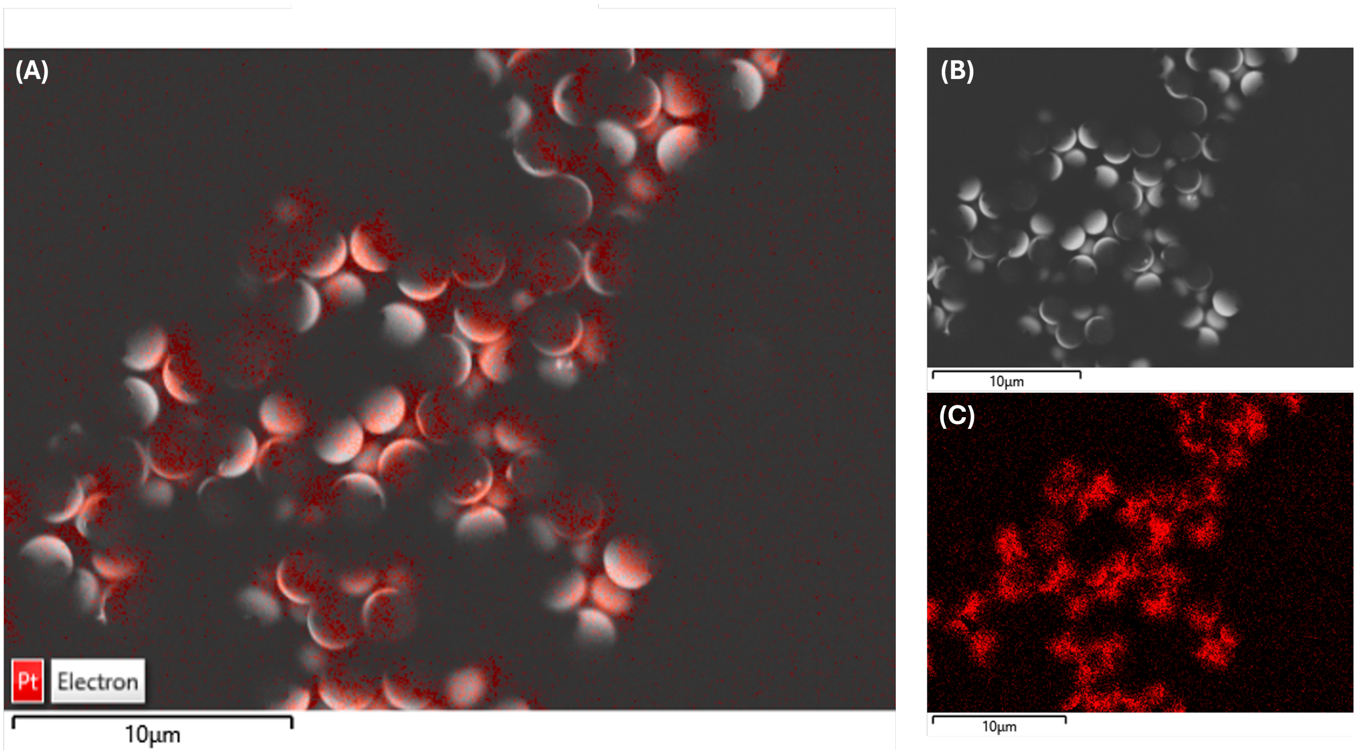

FIG. S2. (a) Overlay of backscattered electron (BSE) image and EDS elemental map showing the distribution of Pt (red). (b) BSE image of the particles, where the higher contrast arises from regions with higher atomic number. (c) Corresponding EDS elemental map confirming the presence of Pt, consistent with the observed contrast in the BSE image.

film forming phase surrounding the particles. Compared to the bright platinum regions, the film forming matrix appears uniformly darker due to its lower atomic number. Across some regions, (Figure S3)it is clearly observed that the film forming particles have coalesced into a continuous overlayer during drying, which can hinder the determination of the JC orientation. These particles were included in the overall count but grouped within the non-resolved fraction whenever their orientation could not be determined unambiguously. The orientation analysis was performed using five independent BSE micrographs collected from three independently prepared coating samples, corresponding to approximately 1000 Janus colloids analysed in total. The reported uncertainties correspond to the standard deviation across the five independently analysed BSE images.

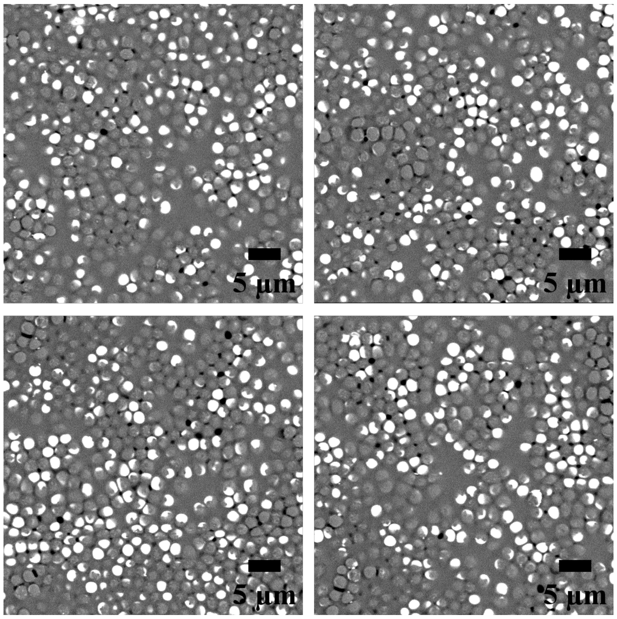

FIG. S3. Backscattered electron (BSE) micrographs of the dried films. The bright regions correspond to the platinum-coated hemispheres of the JCs. In some regions of the film, a darker layer is visible adjacent to the particle surfaces, indicating that the film forming particles locally covers portions of some particles during film formation.

II. SIMULATION DETAILS

Unless stated otherwise we performed simulations by discretising the dimensionless Equations (4)-(6) using the Euler-Maruyama method with a timestep of $\Delta t = 5 \times 10^{-7}$. We initialize our simulations with a mix of 400 big particles and 20 000 small particles randomly distributed in a 100×100 sized box with periodic boundary conditions in the x-direction.

We introduce a dimensionless sedimentation speed

$\tilde{v}_{\rm g} = v_{\rm g}\sigma_{\rm b}/D_{\rm b}$. The rate of evaporation is characterized by $\tilde{v}_{\rm interface} = v_{\rm interface}\sigma_{\rm b}/D_{\rm b}$. Unless reported otherwise simulations are performed with $\tilde{v}_{\rm interface} = 1$, a size ratio $\tilde{\sigma} = 5$ and with big particles sedimenting with $\tilde{v}_{\rm g} = 0.5$.

We ensure the normalization of $\hat{\boldsymbol{e}}_{{\rm b},\alpha}$ using a Lagrange multiplier.

In other circumstances big passive particles may get trapped at the moving interface through surface tension, hydrophobicity, and Pickering effects. We see no evidence of significant numbers of passive big particles at the top surface in the performed experiment (see Figure 3). Therefore we chose to model the air-solvent interface as purely repulsive, as we assume particles to not penetrate the air solvent interface. This enables an isolated analysis of the effect of activity.

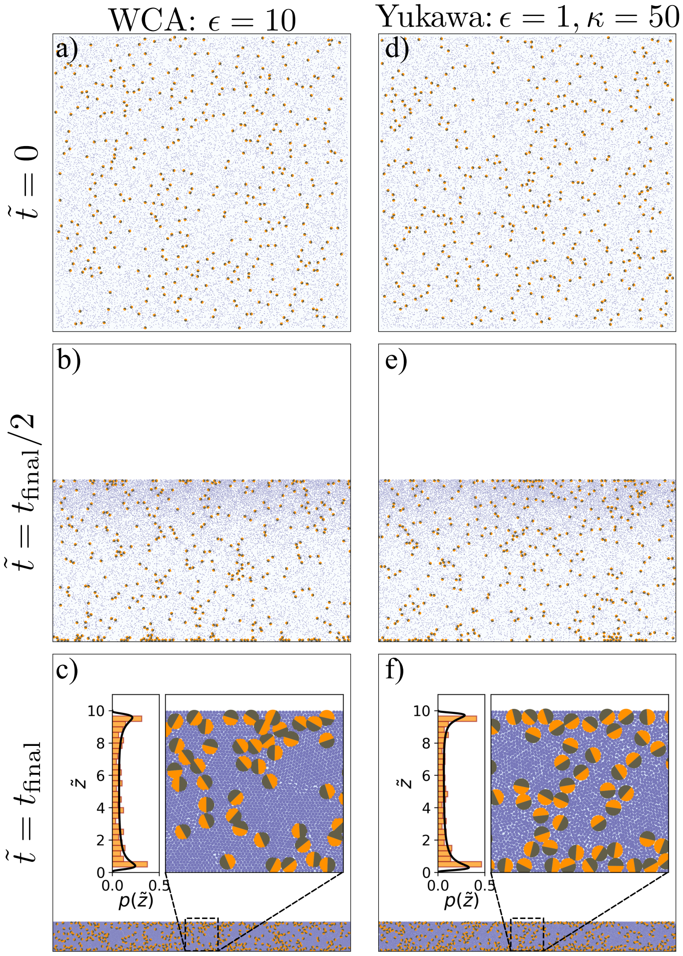

FIG. S4. Time evolution of the simulated systems with different pair potentials. A zoom into the final film configuration is shown as an inset. The accompanying histograms show the vertical distribution of JCs. The left column shows a simulation utilizing a stiffer WCA potential compared to simulations shown in Figure 5 with $\epsilon = 10$. The right column shows a simulation utilizing a short range Yukawa potential (see Eq. S5) with $\epsilon = 1$ and $\kappa = 50$. Both simulations show distributions of JCs similar to those shown in Figure 5. For stiffer interaction potentials, small particles show stronger hexagonal ordering.

A. Interaction potentials

In our simulations particles interact through a potential $\tilde{U}$ with each other and interfaces

$$\tilde{U} = U_{\rm bottom} + U_{\rm top}(t) + \sum_{\alpha<\beta} U_{\alpha\beta}(r_{\alpha\beta}) \qquad \text{(S1)}$$

Here the sum $\sum_{\alpha<\beta}$ denotes a sum over all particle pairs (irrespective of size) and $r_{\alpha\beta} = |\tilde{\boldsymbol{x}}_\beta - \tilde{\boldsymbol{x}}_\alpha|$ the interparticle distance. We assume particles to interact only through steric repulsion and to be completely hydrophilic and not penetrate the solvent-air interface. For simplicity and computational efficiency we use the repulsive part of a Lennard-Jones potential, known as the Weeks-Chandler-Andersen (WCA) potential [2], for all these interactions. The pair potential $U_{\alpha\beta}$ may be written as

$$U_{\alpha\beta} = \begin{cases} \epsilon\left[\left(\frac{\sigma_{\alpha\beta}}{r_\alpha}\right)^{12} - 2\left(\frac{\sigma_{\alpha\beta}}{r_{\alpha\beta}}\right)^{6}\right] + \epsilon & \text{if } r < \sigma_{\alpha\beta} \\ 0 & \text{else} \end{cases}, \qquad \text{(S2)}$$

with a size dependent interaction range $\sigma_{\alpha\beta} = (\sigma_\alpha + \sigma_\beta)/2$. Here σ_α and σ_β are the dimensionless diameters of the interacting particles. Similarly contribution of bottom interface may be written as

$$U_{\rm bottom} = \begin{cases} \epsilon\left[\left(\frac{\sigma_\alpha}{2\tilde{z}_\alpha}\right)^{12} - 2\left(\frac{\sigma_\alpha}{2\tilde{z}_\alpha}\right)^{6}\right] + \epsilon & \text{if } \tilde{z}_\alpha < \frac{\sigma_\alpha}{2} \\ 0 & \text{else} \end{cases}, \qquad \text{(S3)}$$

and that of the top interface as

$$U_{\rm top} = \begin{cases} \epsilon\left[\left(\frac{\sigma_\alpha}{2(h(\tilde{t})-\tilde{z}_\alpha)}\right)^{12} - 2\left(\frac{\sigma_\alpha}{2(h(\tilde{t})-\tilde{z}_\alpha)}\right)^{6}\right] + \epsilon & \\ & \text{if } h(\tilde{t}) - \tilde{z}_\alpha < \frac{\sigma_\alpha}{2} \\ 0 & \text{else} \end{cases}, \qquad \text{(S4)}$$

with the time dependent position of the solvent-air interface $\tilde{z}_{\rm top}(t)$. Unless stated otherwise we choose $\epsilon = 1$ as the strength of the repulsive potentials.

B. Influence of the pair-potentials

We find that the reported results do not depend on the exact shape of the pair-potential, as long as it is purely repulsive and sufficiently hard. We chose the previously described WCA potential for computational efficiency, but find that the nature of our results does not depend on the exact value of ϵ nor the exact shape. Simulations performed with a value of $\epsilon = 10$ and a short range Yukawa interaction used for similar systems [3]

$$U_{\alpha\beta} = \begin{cases} \epsilon\exp(-\kappa(r_{\alpha\beta} - \sigma)) & \text{if } r_{\alpha\beta} < (\sigma_\alpha + \sigma_\beta)/2 + \sigma_{\rm small} \\ 0 & \text{else} \end{cases}, \qquad \text{(S5)}$$

with $\epsilon = 1$ and $\kappa = 50$ are shown in Figure S4.

C. Particle size ratio

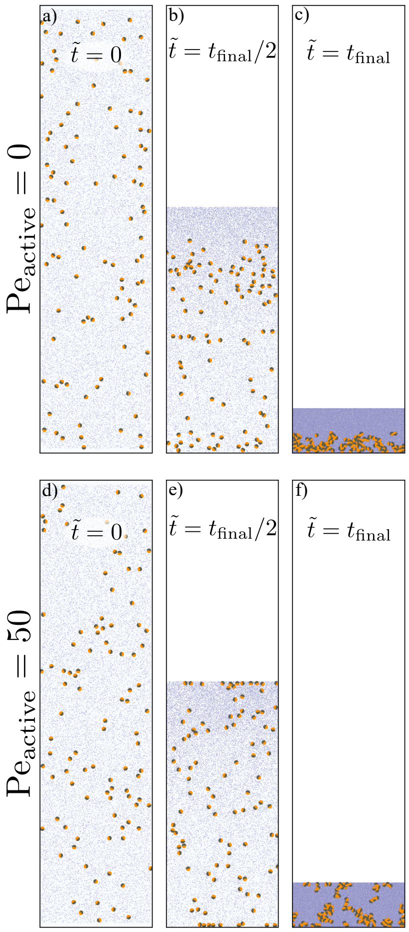

FIG. S5. Simulations performed with a size ratio of $\tilde{\sigma} = 10$. The top row (a,b,c) shows a simulation with $Pe_{\text{active}} = 0$, and the bottom row (d,e,f) with $Pe_{\text{active}} = 50$. The number density of small particles has been quadrupled to achieve comparable area fractions. Simulations are performed with a time step $\Delta t = 10^{-7}$ to account for faster particle diffusion. Because of increased number density and decreased time step the simulation domain has been narrowed to $25\sigma_{\text{b}}$ to keep the computational effort feasible.

We chose to perform simulations with a relative particle size of $\tilde{\sigma} = 5$ for computational efficiency. Smaller particles possess a higher diffusion constant D_{s} requiring a smaller timestep Δt as well as a higher number of particles to achieve the same volume fraction. Simulating with a larger size ratio $\tilde{\sigma}$ did prove prohibitive with the available computational resources. To investigate the effect of relative particle size we performed simulations with $\tilde{\sigma} = 10$ (see Figure S5) closer to the experimentally measured values. We observe chemical gradients driving big particles downward to be stronger with bigger $\tilde{\sigma}$, but particles with sufficient activity can still overcome the auto-stratification effect and the final active particle distribution is analogous to that observed in the $\tilde{\sigma} = 5$ system.

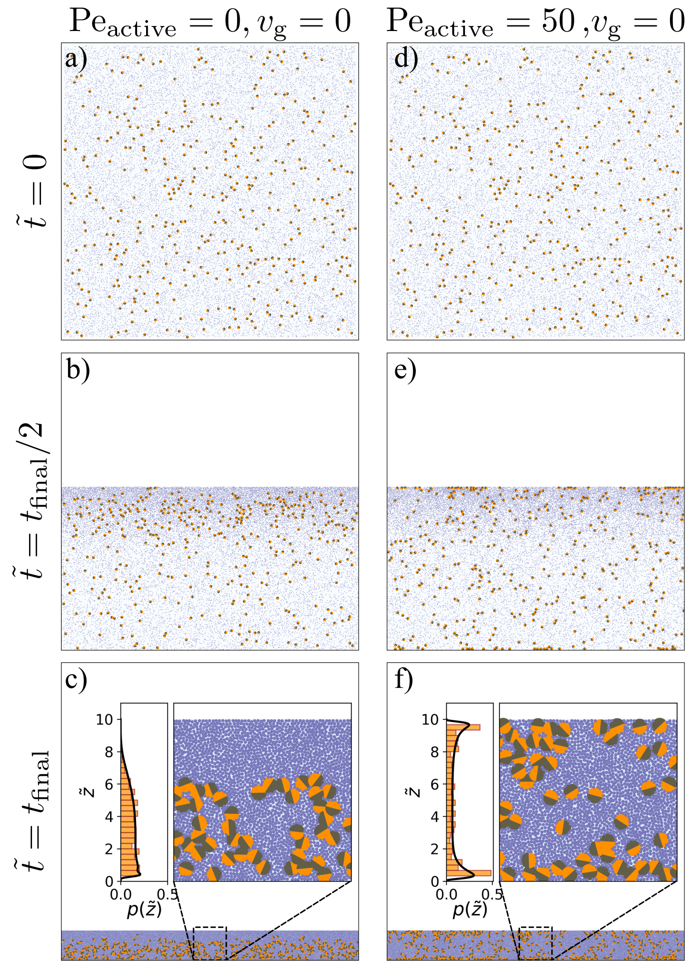

FIG. S6. Time evolution of the simulated system in the absence of gravity $v_{\text{g}} = 0$ for passive (left) and active (right) colloids. A zoom into the final film configuration is shown as an inset. The accompanying histograms show the vertical distribution of JCs. In the passive case ($Pe_{\text{active}} = 0$) JCs are still expelled from the top interface completely, but the absence of gravity leads to a flatter distribution compared to the $v_{\text{g}} = 0.5$ case shown in Figure 5. The distribution in the active case ($Pe_{\text{active}} = 0$) is almost indistinguishable to the one with gravity, as activity is the dominant effect in both cases $Pe_{\text{active}} \gg v_{\text{g}}$.

D. Influence of gravity

We include a slight negative buoyancy into our model equations resulting in a sedimentation speed v_{g}, as the JCs used in experiments have slight negative buoyancy too. However, the nature of our results does not critically depend on this negative buoyancy as the chemical potential gradients generated by the small particle distribution

is enough to drive the JCs away from the liquid-gas interface. Simulations showing this effect in the absence of gravity $v_\mathrm{g} = 0$ are shown in Figure S6.

E. Influence of small particle density

The small passive particles are responsible for a gradient in the chemical potential that drives the larger colloids away from the moving interface in the absence of activity. To investigate the importance of this effect we perform simulations with varying number of small particles. All other simulations have an initial number density of $\rho_0 = 2$ that increases as the system shrinks. Figure S7 shows simulations with different numbers of small particles (while the number of large colloids is kept constant). An increasing number of small particles promotes the expulsion of JCs from the top interface. In the case with no small particles in the system $\rho_0 = 0$ big colloids are found with an almost uniform distribution, as their sedimentation is slower than the speed of the moving interface. Active particles show bimodal distribution, with slightly higher peaks at the interfaces, as their movement is not impeded by the small ones.

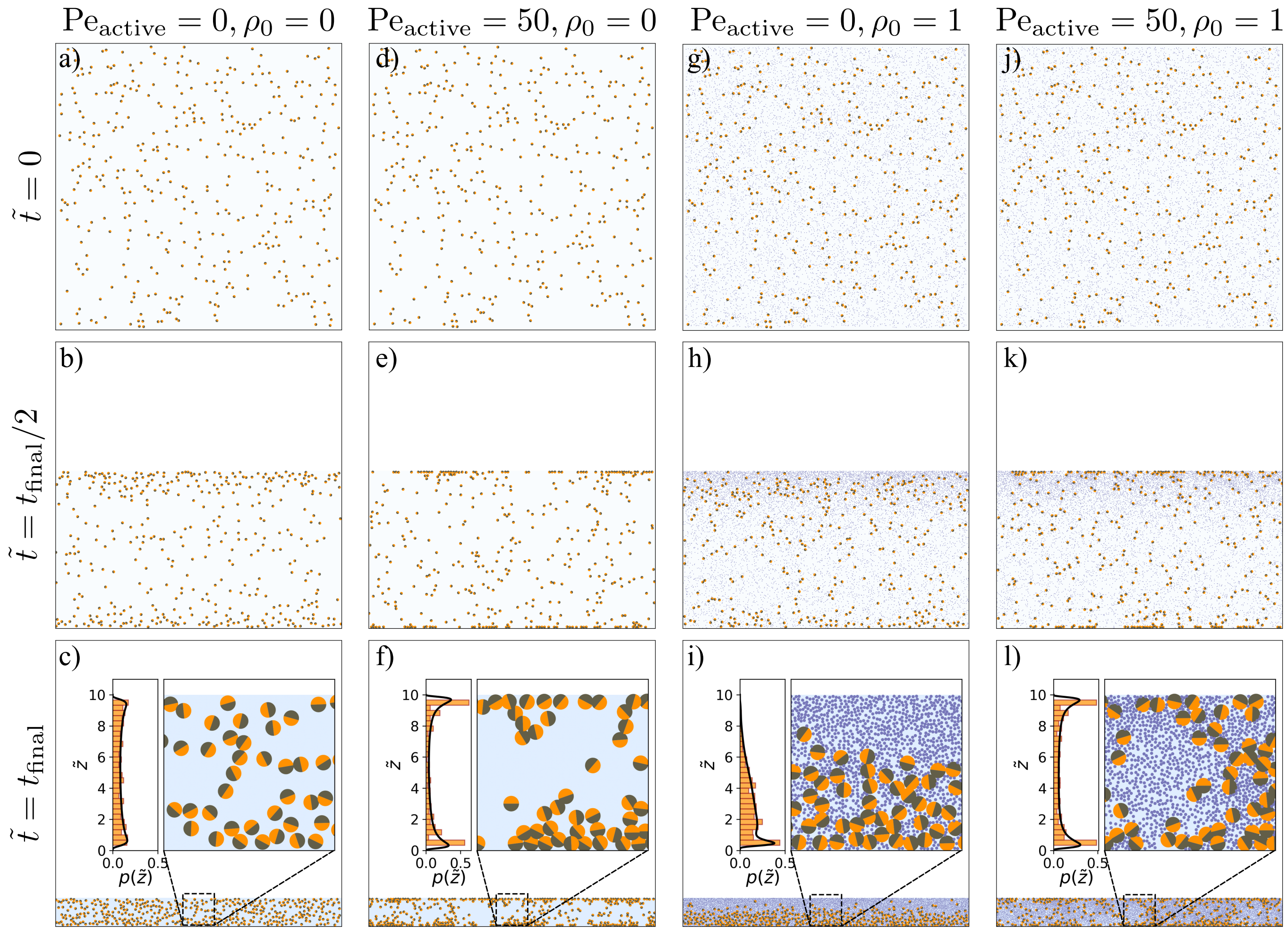

FIG. S7. Time evolution of the simulated with different initial number densities of small particles ρ_0. A zoom into the final film configuration is shown as an inset. The accompanying histograms show the vertical distribution of JCs. In the passive case ($Pe_{\mathrm{active}} = 0$) JCs are not expelled from the top interface in the absence of small particles (column 1), or the effect is weaker (column 3) when the density of small particles is lower compared to the simulations shown in Figure 5. Active particles always show wall-hugging behavior with slightly higher probability density peaks at the walls in the absence of small particles (column 2) compared to systems with small passive particles (column 4).

F. Kernel density estimation of bounded distributions

To estimate vertical probability density functions $p(\tilde{z})$ we use a Kernel Density Estimation (KDE) method [4]. KDEs provide an effective tool to estimate a density function from a finite number of samples. However, the physics of the system dictate that the probability density function is bound to the space between the top and bottom interface, such that $p(\tilde{z}) = 0$ for $\tilde{z} \notin (0, h(\tilde{t}))$. KDE methods ar known to struggle estimating bounded distributions predicting nonzero values of probability density outside the bounding interval [5]. To address this problem we transform our sampled coordinates using a logit transform to span the whole real axis such that

$$\hat{z} = f(\tilde{z}) = \log\left(\frac{\tilde{z}/h}{1 - \tilde{z}/h}\right) . \tag{S6}$$

We then perform a standard KDE of the unbounded distribution of $\hat{p}(\hat{z})$ with a bandwidth given by $\left(\frac{4\sigma_{\hat{z}}^5}{3n}\right)^{1/5}$ [6], where $\sigma_{\hat{z}}$ is the standard deviation of the samples and n the number of samples. We then obtain $p(\tilde{z})$ through a transformation back to the bounded interval

$$p(\tilde{z}) = \hat{p}(\hat{z})|f'(\tilde{z})| \, . \tag{S7}$$

Such transformations are a common tool to estimate bounded probability density distributions [5, 7].

III. SUPPLEMENTARY VIDEOS

Video S1: Particle motion after 1 hour of adding the fuel.

[1] E. Velasquez, J. Rieger, F. Stoffelbach, F. d'Agosto, M. Lansalot, P.-E. Dufils, and J. Vinas, Surfactant-free poly (vinylidene chloride) latexes via one-pot {RAFT}-mediated aqueous polymerization, Polymer **106**, 275 (2016).

[2] J. D. Weeks, D. Chandler, and H. C. Andersen, Role of repulsive forces in determining the equilibrium structure of simple liquids, The Journal of Chemical Physics **54**, 5237 (1971).

[3] A. Fortini, I. Martín-Fabiani, J. L. De La Haye, P.-Y. Dugas, M. Lansalot, F. D'agosto, E. Bourgeat-Lami, J. L. Keddie, and R. P. Sear, Dynamic stratification in drying films of colloidal mixtures, Physical Review Letters **116**, 118301 (2016).

[4] E. Parzen, On estimation of a probability density function and mode, The Annals of Mathematical Statistics **33**, 1065 (1962).

[5] G. Geenens, Probit transformation for kernel density estimation on the unit interval, Journal of the American Statistical Association **109**, 346 (2014).

[6] B. W. Silverman, *Density estimation for statistics and data analysis* (Routledge, 2018).

[7] G. Geenens and C. Wang, Local-likelihood transformation kernel density estimation for positive random variables, Journal of Computational and Graphical Statistics **27**, 822 (2018).
